# Exploring Mental Health Barriers Among At-Risk Adolescents: An Integrative Analysis of Self-Reports and School Nurses’ Perspectives

**DOI:** 10.3390/bs16050833

**Published:** 2026-05-21

**Authors:** Minjeong Kim, Seolhyang Baek

**Affiliations:** College of Nursing, Dongguk University WISE Campus, Dongdaero 123, Gyeongju 38066, Republic of Korea; kjmmjk7@gmail.com

**Keywords:** at-risk adolescents, mental health barriers, mental health stigma, somatization, self-harm, school nurses

## Abstract

In modern society, adolescents experiencing mental health problems are increasing. This study aims to identify barriers to mental health care at the individual, family, school, and staff levels among at-risk adolescents, employing a mixed-methods approach. Given the ethical and practical constraints of engaging at-risk adolescents directly, the study quantitatively analyzed responses to the AMPQ-III-I survey from 47 runaway adolescents, while conducting interviews with eight school nurses serving as proxy informants. The at-risk adolescents were found to be in a state of mental health crisis characterized by somatization, self-harm, excessive digital media use, and peer imitation. Within the family environment, they experienced communication gaps with adults, concerns about mental health stigma, and the risk of disengagement from home and school. Despite experiencing physical and emotional difficulties that hindered their ability to focus on academic work, schools tended to deprioritize mental health, and these adolescents reported notably low utilization of professional counseling. School nurses, although well-positioned to identify at-risk adolescents, expressed barriers such as excessive workload and a lack of communication among teachers. These findings suggest that, to support the growing and intensifying population of at-risk adolescents, an urgent shift in awareness and the alleviation of barriers within the family–school–staff ecosystem is required.

## 1. Introduction

Globally, mental health issues among adolescents are on the rise, emerging as a major global health burden. As of 2023, an estimated 5.3 million adolescents aged 12 to 17, constituting approximately 20.3% of this age group, were reported to be afflicted with anxiety, depression, or behavioral/conduct problems. This figure signifies a substantial increase of approximately 35%, which is notably higher than the 15.0% prevalence rate of adolescent mental health problems that was documented in 2016 (Sappenfield et al., 2024). Similarly, in Korea as of 2024, the prevalence of perceived stress among adolescents over the past three years increased from 37.3% to 40%, and the prevalence of depression was also reported to be over 25% (Korea Disease Control and Prevention Agency, 2025). According to the 2021 Nationwide Adolescent Mental Health Survey in South Korea, suicidal ideation accounted for 18.7%, with depressive symptoms at 11.8%, somatic symptoms at 57.9%, and self-harm behaviors at 27.4% (J. Kim & Cho, 2024). In response to this situation, the World Health Organization (WHO) has designated child and adolescent mental health as a core public health priority and is promoting a comprehensive mental health promotion strategy (WHO, n.d.). South Korea also incorporated mental health management into the scope of school health through the 2006 revision of the School Health Act and has promoted initiatives such as the expansion of a nationwide school-based mental health screening program using the Adolescent Mental Problem Questionnaire (AMPQ) (E. H. Kim & Kim, 2016; S. J. Kim et al., 2009).

Despite such policy endeavors, the prevalence of adolescent mental health problems continues to grow, suggesting that the issue cannot be adequately understood through individual-level pathology alone. For instance, low awareness of mental health and concerns about social stigma have been identified as significant barriers that delay adolescents’ help-seeking and early intervention (Rickwood et al., 2005; Schnyder et al., 2017), yet the way such stigma is jointly constructed by adolescents, parents, and school personnel has rarely been examined in an integrated manner. Likewise, somatization is well documented as a common channel through which adolescents express emotional distress that they cannot articulate verbally (Lu et al., 2025; Mahirah et al., 2025), but its role as an early signal that intersects with the school’s organizational handling of physical and mental health remains underexplored. In addition, the rapid expansion of digital media use in contexts where home-based caregiving is constrained has introduced a novel pathway through which crisis behaviors may be learned and reinforced. Zhang et al. (2025) demonstrated that parental neglect is significantly associated with adolescents’ social media addiction, with this relationship sequentially mediated by unmet basic psychological needs and diminished personal growth initiative (Zhang et al., 2025). At the same time, Wanniarachchi et al. (2025) identified concerns about stigma, privacy, and trust as persistent barriers that limit adolescents’ meaningful engagement with digital mental health services (Wanniarachchi et al., 2025). When such ecological barriers go unaddressed, adolescents may gradually disengage from the protective systems of home and school (H. R. Kim & Moon, 2023). In line with this, Castillo et al. (2023) reported that running away is a more widespread phenomenon than is often assumed, showing strong associations with family conflict, school maladjustment, peer difficulties, and mental health problems (Castillo et al., 2023). Once adolescents have disengaged from such protective networks, the consequences tend to compound; Prince et al. (2019), drawing on a national sample of youth transitioning out of foster care, found that adverse outcomes such as homelessness, substance use, and deteriorating mental health become increasingly likely among this population (Prince et al., 2019).

Conversely, the extant research on at-risk adolescents predominantly comprises secondary analyses of large-scale data sets (Castillo et al., 2023; H. R. Kim & Moon, 2023; J. Kim & Cho, 2024; Prince et al., 2019) or studies that concentrate on a solitary risk factor (Martinez-Casanova et al., 2024; Schnyder et al., 2017). Moreover, it is not easy in practice to directly include at-risk adolescents as participants in research due to ethical considerations, difficulties in obtaining parental consent, and limitations in accessibility (Askari et al., 2024; Sikand et al., 1997).

Within the school context, school nurses occupy a distinctive position: they are often the first to detect changes in students’ daily behavior and early signs of physical and emotional distress (McDermott et al., 2018; Moen & Jacobsen, 2022; Thomas et al., 2025), and they are routinely consulted by adolescents who are reluctant to approach formal counseling services. This positioning makes them uniquely informative proxy informants regarding the broader population of at-risk adolescents and the structural conditions that shape their access to mental health support.

Whereas at-risk adolescents constitute a broader population whose mental health vulnerabilities unfold along diverse trajectories within school and community contexts, runaway adolescents in this study are conceptualized as a subgroup of at-risk adolescents who represent one of the most acute and visible manifestations of adolescent crisis (Castillo et al., 2023; H. R. Kim & Moon, 2023). Building on this conceptual positioning, the present study was undertaken to provide a multifaceted understanding of how the experiences of runaway adolescents intersect with the contextual realities of at-risk adolescents observed in everyday school settings, and of how ecological barriers manifest within a single continuum of adolescent risk.

## 2. Methods

### 2.1. Design and Ethical Considerations

Adolescents often exhibit reluctance in disclosing sensitive personal experiences and emotional difficulties to external individuals. Particularly in the case of adolescents in crisis, it is extremely difficult to obtain sufficient information during interviews. A qualitative approach that utilizes proxy informants, who are experts familiar with adolescents and capable of integrating information from multiple sources, is often recommended to compensate for these limitations (Kisiel et al., 2014). Given these grounds, this study adopted a mixed-methods approach, which involved conducting a simple direct survey with at-risk adolescents while concurrently employing a qualitative approach using proxy informants instead of having the participants directly engage in interviews.

This study was approved by the Dongguk University WISE Institutional Review Board (IRB Number: DGU IRB 202500004-01), and all study procedures adhered to research ethics principles designed to protect the human rights and safety of vulnerable participants (World Medical Association, 2025).

### 2.2. Quantitative Study

#### 2.2.1. Participants

Runaway adolescents are reported to primarily enter temporary youth shelters operated by local governments to receive short-term protection (Ministry of Gender Equality and Family, 2025). This study recruited runaway adolescents residing at a temporary youth shelter located in Busan Metropolitan City as study participants. Participation in the study was limited to adolescents aged 14 years or older who received a thorough explanation of the purpose and procedures of the research and provided informed consent to participate. Based on this age criterion, parental consent was waived (Santelli et al., 2025). The final sample size for this study was 47 participants.

#### 2.2.2. Instruments

This study employed the Adolescent Mental Problem Questionnaire, Third Version with Improvement (AMPQ-III-I) as the assessment instrument. The instrument includes domains assessing personality traits and emotional and behavioral characteristics, including suicide risk (Korea Educational Environment Protection Agency, 2025). Each item in the original instrument is scored on a 4-point scale ranging from 0 to 3 points.

#### 2.2.3. Data Collection and Analysis

The data collection period spanned from 1 May to 31 July 2025, focusing on the aforementioned 50 runaway adolescents. After excluding three sets of data with low response reliability from the collected materials, data from a total of 47 adolescents were used for the final analysis.

To enable an integrated qualitative interpretation of the mental health status of runaway adolescents, this study analyzed only the 36 items pertaining to the emotional and behavioral characteristics and suicide risk domain. The personality traits domain was excluded from the main analysis and presented in Appendix A, as the personality subscale is primarily designed for use in counseling settings and does not provide cut-off points, which was deemed inconsistent with the analytic objectives of the present study.

Although the emotional and behavioral characteristics and suicide risk domain in the original instrument comprises five subscales along with suicide risk items, this study did not analyze the data based on subscale-level scores. Instead, the analysis focused on the response patterns of individual items. This item-level approach was adopted to capture the specific manifestations of symptoms and crisis behaviors among runaway adolescents and to derive themes through qualitative integration of the response patterns.

For analysis, the original 4-point response format was recoded into a dichotomous “yes/no” format to identify the presence or absence of each symptom and crisis behavior. The quantitative data were analyzed using descriptive statistics to calculate the frequency and percentage of responses for each item. Based on these results, similarities in response patterns were identified, and the findings were qualitatively integrated to derive themes.

### 2.3. Qualitative Study

#### 2.3.1. Participants

A purposive sampling method was used to identify participants with experience supporting at-risk or runaway adolescents among school nurses working in middle and high schools within Busan Metropolitan City and Gyeongsangbuk-do (Palinkas et al., 2015). Subsequently, to expand the sampling scope, a snowball sampling method was employed in parallel. Participants were briefly informed of the purpose of the study through referrals from initial participants, and those expressing willingness to participate were selected as final participants. Following this procedure, eight school nurses were finally selected as participants in the study.

#### 2.3.2. Instruments

This study developed an in-depth interview guide for school nurses to understand the ecological processes through which crisis behaviors emerge and unfold among adolescents. First, a preliminary overview was established through a review of prior literature on at-risk adolescents. Subsequently, after consulting with one nursing professor and one social welfare professor with experience supporting at-risk adolescents, the content validity and applicability of the items were reviewed, culminating in the finalization of the in-depth interview guide. The in-depth interview guide consisted of a total of 10 items, including questions such as “What emotional or behavioral problems might at-risk adolescents exhibit?” (Appendix B).

#### 2.3.3. Data Collection and Analysis

Data were collected from 14 May to 4 July 2025, and interviews were conducted in health offices at each school for approximately 90 min. All interviews were audio-recorded with the consent of participants, and the recorded data were transcribed and utilized for analysis.

The transcribed interview data were analyzed following Braun and Clarke’s six-phase thematic analysis procedure (Braun & Clarke, 2006): (1) familiarization with the data through repeated reading, (2) generating initial codes through line-by-line coding, (3) searching for themes by clustering related codes, (4) reviewing and refining themes through constant comparison across transcripts, (5) defining and naming themes, and (6) producing the final analytic narrative. Two researchers independently coded each transcript, and discrepancies were resolved through repeated discussion until consensus was reached. Memo writing was used throughout the process to record analytic decisions, emerging interpretations, and potential biases, thereby supporting reflexivity and an audit trail.

Ensuring trustworthiness is crucial in establishing the credibility and reliability of qualitative findings, including elements such as credibility, transferability, dependability, and confirmability (Ahmed, 2024). These criteria were ensured through prolonged data engagement, peer debriefing, member checking, thick description, and an audit trail reviewed by an external researcher.

## 3. Results

### 3.1. Quantitative Findings

#### 3.1.1. General Characteristics of the Participants

Of the 47 runaway adolescents included in the final analysis, 15 (31.9%) were males and 32 (68.1%) were females. The majority were high school students, totaling 33 (70.2%), while 14 (29.8%) were middle school students. The age of participants ranged from 14 to 18 years old, with an average age of 16.2 years (not presented in the table).

#### 3.1.2. Results of the Emotional and Behavioral Assessment

The analysis of responses in the emotional and behavioral characteristics domain, including suicide risk, of the AMPQ-III-I identified three main themes: difficulties in school life and interpersonal relationships, somatic and related symptoms, and crisis behaviors (Table 1). The detailed findings for each theme are as follows.

First, in the area of school life and interpersonal relationships, 68.1% (*n* = 32) reported difficulty concentrating during classes, studying, or reading for a long time. In addition, 12.8% (*n* = 6) reported serious violations of school rules, such as skipping school or running away from home. Among runaway adolescents, 57.4% (*n* = 27) reported becoming irritated when adults instructed them to do things, 38.3% (*n* = 18) reported feeling as if others knew what they were thinking, and 34.0% (*n* = 16) reported feeling that others were talking about them behind their back.

Second, in the domain of somatic and related symptoms, 46.8% (*n* = 22) reported experiencing physical symptoms such as headaches, abdominal pain, nausea, or dizziness without a clear cause. Sleep difficulties, including difficulty falling asleep or waking frequently during the night, were reported by 40.4% (*n* = 19), and 23.4% (*n* = 11) reported hearing something that others could not hear.

Third, in the domain of crisis behaviors, 12.8% (*n* = 6) reported a history of self-harm. Suicidal ideation was reported by 19.1% (*n* = 9), and 10.6% (*n* = 5) reported having made a detailed plan to kill themselves. Additionally, 17.0% (*n* = 8) reported feeling that their death would improve their situation. Difficulty handling daily tasks due to excessive use of the internet, games, or smartphones was reported by 44.7% (*n* = 21), and 21.3% (*n* = 10) reported having consumed alcoholic drinks. Furthermore, 25.5% (*n* = 12) reported having received professional counselling.

### 3.2. Qualitative Findings

#### 3.2.1. General Characteristics of the Participants

The participants in this study consisted of a total of eight school nurses, all of whom were females. The participants ranged in age from 42 to 58 years old, with varying educational levels from an associate’s degree to a master’s degree. Their work experience ranged widely from a minimum of 2.5 years to a maximum of 36.3 years. The types of schools where teachers worked included three general middle schools, three general high schools focused on university admission, and two specialized high schools focused on vocational and career training. School sizes ranged from 169 to 590 students, encompassing diverse school environments surrounding adolescents (Table 2).

#### 3.2.2. Results of the Interview Analysis

Theme 1. Worsening Adolescent Emotional and Behavioral Problems

School nurses have indicated that issues involving at-risk adolescents are no longer confined to specific schools or age groups; rather, they are increasingly manifesting in severe forms within the multi-layered and complex relationships between family, school, and community. For instance, it has been noted that rather than a singular crisis manifesting in isolation, multiple emotional and behavioral problems often co-occur and recur repeatedly.

*“In a class of about 100 students, there are typically two or three children who are particularly serious cases among those considered at risk, and about five children who seem ordinary but have some minor issues. Some kids seem to be struggling to adjust to school, and the number of children who don’t want to come to school or study is increasing…”*

(SN8)

This finding suggests that the increasingly intricate and pervasive challenges faced by adolescents are indicative of a societal phenomenon that cannot be attributed to the responsibility of specific entities, such as parents or schools. Furthermore, crisis situations of this nature underscore the limitations of rigid school operational manuals and existing response systems in addressing such situations in a sufficient manner.

*“I can understand the stance of the school administrators. If there are only one or two at-risk students among the entire student body, like within 5% of the total, I think it would be manageable by an administrator. However, if the number keeps increasing and incidents keep piling up, it would be overwhelming.”*

(SN3)

*“High school students have enough discernment to recognize the principle as such when things are done properly, but middle schoolers are just not mature enough. Young students may feel less attached to school when we follow the manual. But if we follow the manual, the kids go home and say, ‘Our teacher is scary,’ and file complaints.”*

(SN5)

Theme 2. Somatic Symptoms as a Means of Expressing Emotional Distress

School nurses reported that students in crisis often visit the school nurse’s office citing physical symptoms such as headaches, stomachaches, or breathing difficulties as excuses, rather than directly expressing their inner pain, emotional difficulties, or emptiness. A considerable number of cases that were reviewed by the school nurse’s office revealed no discernible medical abnormalities. However, it was observed that students who made multiple visits or requested extended absences frequently exhibited concomitant emotional difficulties.

*“I suspect that when there’s something lacking, it manifests as somatization, leading to frequent visits to the school nurse’s office. Children often lack the courage to fully open up to their counselor about their emotional struggles… Of course, in an environment lacking care, they might actually have stomachaches, and since they’re always struggling, they might also have frequent headaches. Children seem to visit the school nurse’s office first to express such a state, to express that they are in pain!”*

(SN5)

*“We have nearly 170 students, and about 30 of them visit the school nurse’s office every day. How many students among them do you think are sick? There are no students who are actually sick. Almost none… I think a lot of those kids come because they need attention and have emotional pain. Children who frequently ask to be put to bed or request headache medicine often seem to be those with deep emotional wounds. When I say things like, ‘You take headache medicine too often, don’t you?’ or ‘You try to sleep in the school nurse’s office too often, don’t you?’ they often burst into tears.”*

(SN2)

School nurses recognize that for students who frequently visit the school nurse’s office citing nonspecific physical symptoms, careful observation and additional interviews are required to identify their underlying emotional burdens.

*“When students say they have a real headache or stomachache, they don’t actually have a fever. In fact, they’re physically perfectly fine. However, I’ve been keeping a close eye on those kids lately. I worry these kids might eventually develop depression or anxiety…”*

(SN3)

Theme 3. Self-Harming Behaviors as a Means of Expressing Distress

According to school nurses, self-harming behaviors among at-risk adolescents are often a means of externalizing their pain, meaning they tend to be repetitive rather than confined to a single incident.

*“There is a student who comes to the school nurse’s office to take a nap when feeling depressed, making regular visits. I heard that this child constantly engaged in self-harm, resulting in wounds all over the body during the first year. When I asked, ‘Why did you harm yourself?’ the child explained that it was because the pain was too much to bear. Whenever feeling stressed, the child would cut the arms or back of the hands, saying that drawing blood from the body brings a sense of being alive…”*

(SN2)

Meanwhile, school nurses have noted that self-harm often occurs in households with caregiving gaps or in single-parent families.

*“Students often engage in self-harm at home rather than at school, and marks from self-harm are frequently visible on their arms or thighs. If they cut themselves at school, it would bleed… So they usually cut themselves at home before coming to school. By the time I happen to see the marks on their arms and ask, ‘When did this happen?’ it’s already been a long time since it happened. Kids like that usually come from troubled family backgrounds or have divorced parents… They get so angry during conversations with their father or mother… When they can’t control their rage, they start tearing at their own flesh…”*

(SN3)

Based on statements from school nurses, most self-harm is non-suicidal in nature, serving as a means to relieve emotional distress. However, there is concern that it can occasionally lead to serious injury or even fatal outcomes such as suicide.

*“Cutting wrists, cutting thighs, some kids have scars all over their bodies… There was a student who took two weeks’ worth of medication at once and ended up being rescued by the ambulance… And our school had a suicide incident a few years ago… During the investigation, the body of the girl who committed suicide showed signs of self-harm.”*

(SN2)

Theme 4. Digital Media as a Source of Harmful Exposure

In community settings, school nurses have indicated that as the influence of digital media expands, adolescents are becoming excessively exposed to harmful information and stimuli. These environmental factors act as antecedents, triggering crisis behaviors in adolescents. The impact of exposure to harmful environments, such as violent incidents or online gambling, which are readily accessible to young individuals through the internet and online video platforms, on mental health outcomes, including depression, emotional distress, and addictive behaviors, has been a subject of considerable attention. It has been stated that the influence of these digital media is manifesting as a decline in patience and concentration among adolescents, increased impulsivity, and difficulties in achieving physical and emotional stability.

*“Troubled youth… They seem to be in a serious trouble… Children seem to be easily exposed to various incidents, accidents, and harmful environments through media like computers and YouTube. The age at which children are exposed to harmful content online is also getting younger. For this reason, it seems that more children are showing signs of depression. There are also kids who get stuck in online gambling. Kids these days really have no patience. They indulge in short videos like YouTube Shorts, which has left them lacking in patience and concentration. They can’t even hold the same posture for a minute. Most of the kids are lying down, chatting away beside them…”*

(SN7)

School nurses have expressed concern that insufficient care at home increases the tendency for adolescents to become dependent on their smartphones or social media, potentially leading to crisis behaviors such as self-harm.

*“If they don’t receive sufficient protection at home, they essentially face the same neglect at school. This pattern continues through elementary, middle, and high school. Ultimately, with no one to compensate them (with love and attention) and no one to care for them, they resort to self-harm. When they post this on social media, someone might offer a warm word like, ‘That must be tough,’ or show some concern. This seems to be spreading like a trend among them.”*

(SN3)

Theme 5. Peer Imitation and Reinforcement of Crisis Behaviors

School nurses have observed that at-risk youth tend to imitate risky behaviors influenced by their peers, repeating and reinforcing these actions.

*“Children seem to use social media like Instagram a lot. They post selfies of their self-harm, which seems to be catching on like a trend. When you talk to the children, they say,’ I cut myself,’ without batting an eye. ‘I did it at home, just cut myself with a knife,’ they say calmly.”*

(SN3)

*“A survey of the entire school found that nearly half of the female students showed signs of self-harm. They seem to engage in self-harm like a trend. Seeing friends cutting, they try it themselves. It breaks my heart to see one of the students covered in hypertrophic scars, looking like plump snakes or the stripes of a zebra. When right-handed kids show up wearing a wrist guard on their left arm… it means they have self-harmed their left arm.”*

(SN4)

Concurrently, in the context of peer culture, cases of self-harm have been documented, wherein individuals deliberately inflict harm upon their own bodies. Additionally, there have been reports that crisis behaviors among at-risk youth are mutually accepted and reinforced. School nurses identified peer interactions as a factor contributing to the impairment.

*“They might be imitating TV dramas and movies… This is a world adults just don’t understand… I’m not talking about organized brawls with weapons, but even when it’s just punches being thrown back and forth, they seem to feel absolutely no guilt about it… Students always seem different when they come back after vacation. There are times when kids who were acting out start to improve, and kids who seemed naive and innocent come back with strange new habits after vacation, making you wonder what happened. Kids who do not seem like the type suddenly start smoking or…”*

(SN7)

*“There were kids who got hooked on gaming, ended up smoking and drinking, and even stole motorcycles. They hardly show up at school either. When the kids were together, it was like, ‘What’s the big deal?’ Stealing things from convenience stores or sneaking cigarettes, they just go, ‘What’s the big deal?’ They end up stealing repeatedly, and eventually get reported by other students… Lacking judgment about right and wrong… they still need guidance…”*

(SN8)

Theme 6. Communication Breakdown Between At-Risk Adolescents and Their Parents

School nurses stated that there was a severe lack of care for the overall mental health of adolescents, including a breakdown in parent–child communication, in relation to the home environment of at-risk adolescents. Given this context, cases such as parents’ lack of awareness regarding their duty to provide care or gaps in care due to alcoholism were presented.

*“Parents say they understand, but whether they actually do what they say when we are not around… whether they really act that way toward their children… we can’t really know for sure, can we? The guardians are two-faced. During calls, they say, ‘Right, right’ and ‘I see, I see,’ but in reality, they just neglect it again… The guardians are the problem. Seeing those kids, you realize their parents all have issues too.”*

(SN3)

*“… They are not fulfilling their role as parents yet. Such parents are too busy having fun themselves to care for their children… They just file complaints demanding the school look after their kids, so the school ends up having to pamper the children. School is essentially a daycare center.”*

(SN4)

Theme 7. Absence of Parental Care in At-Risk Adolescents

School nurses stated that in households facing significant livelihood challenges, particularly those with irregular work schedules or night shifts, guardians tend to become desensitized to children’s recurring crisis behaviors. These guardians often demand that schools provide care.

*“Since they’ve been self-harming since middle school or even earlier, their parents are fully aware of it. The patents are just worn out. At first they may have been surprised, but as it happens repeatedly… The parents have jobs, so there’s no way to know how the children are being looked after at home. They don’t seem to provide the kind of one-on-one (meticulous) care you’d expect for kindergarteners.”*

(SN3)

School nurses reported cases where children from busy working families, left unsupervised during parental absence, gathered with peers and engaged in risky behaviors.

*“Households where parents come home late or run a business… especially those open late at night become hangouts for delinquent youth, right? I’ve heard that too. With a mom who works shifts, the child says, ‘My mom’s coming home late! She will not come in tonight! Let’s gather at my place.’ They would go around telling their peers, and then gather at that house in groups, smoking and drinking all night long. So the neighbors would file complaints…”*

(SN7)

Theme 8. Parents’ and Adolescents’ Reluctance Due to Mental Health Stigma

School nurses observed that both parents and adolescents tended to avoid psychiatric counseling and treatment due to a deep-rooted stigma surrounding mental health issues in South Korean society. When students were identified as needing further psychiatric evaluation, parents frequently reacted with denial or anger, refusing to accept their child’s emotional difficulties and resisting referrals to specialized care.

*“Many parents get angry right away. ‘My child is fine. The test must be wrong. What are you talking about?’ Apparently, there are a lot of parents like that. This makes it really hard to recommend counseling.”*

(SN7)

*“When I mention, ‘It seems the child is experiencing severe depression,’ parents sometimes respond, ‘Are you calling my child mentally ill?’ It’s not easy to bring this up. The school believes that the student should receive help (medication) without question, but because of the parents’ misconception that once you start taking psychiatric medication, you have to take it for life, it is really difficult to talk about it directly.”*

(SN2)

A similar reluctance was evident among students themselves, who feared being judged or labeled by their peers if they were seen using counseling services. As a result, even adolescents who recognized their own need for help often hesitated to seek it.

*“One student said nothing was fun or motivating, feeling too skinny… ‘Every time my friends tell me to eat something, it stresses me out a little… I thought about getting counseling, but I can’t go because I’m afraid my friends will think it’s weird if they find out I’m seeing a counselor.’ The student said, ‘I would like to get counseling, but I can’t bring myself to go.”*

(SN3)

Theme 9. Disengagement from Home and School in At-Risk Adolescents

School nurses express deep regret that neglected adolescents at home exhibit crisis behaviors like self-harm, eventually attempt to run away from home, and though they later experience support from school or the community, the underlying issues remain unresolved. They worry these adolescents will disappear from the teachers’ view after running away.

*“The girl was raised in a home where her single mom drank, smoked, and abused her daughter… a home where no care was provided at all. The student returns to such a home, the same situation repeats itself, and the student runs away over and over again…”*

(SN1)

*“Students who engage in self-harm also turn out to be in need of care but are left alone at home. That’s why they keep having those thoughts. They just keep doing it over and over… However, mothers just give their children (psychiatric) medication. It seems like parents just give birth to their children but don’t actually raise them. Eventually, they either drop out of school or go down the wrong path…”*

(SN8)

School nurses also indicated that the standardized framework of school-level support often fails to address the root causes of adolescents’ distress. Repeatedly encountering this gap between the help offered and the help they actually need, students gradually disengage from counseling and, eventually, from school itself.

*“Since we cannot have a deep conversation… Some kids open up completely, but others already know that even if they talk about their emotional pain, nothing will change… because of all the problems at home (we can’t fix that for them, right?) It’s really frustrating. There are many things beyond their control, aren’t there? Well, that’s probably why they don’t go to counseling sessions anymore… These students may have tried everything… Since there has been no significant change since middle school… Some students feel reluctant to attend counseling. Even if I tell them, it just feels like my story will spread everywhere. Still, it seems pretty difficult for the counselor to change that situation either.”*

(SN3)

Theme 10. Grade-Centric School Culture

School nurses have noted that the examination-focused, grade-obsessed school culture that fosters peer competition not only contributes to emotional deprivation but also poses a significant threat to students’ mental health, potentially resulting in panic disorders, school dropouts, and even suicide attempts.

*“…Since some students experiencing high academic stress develop panic disorder, we are currently referring them to the Department of Psychiatry for medication therapy.”*

(SN1)

*“This student didn’t come to the school nurse’s office often either. One day, the kid said, ‘Just a moment, teacher,’ waiting until the other students had left. When the class bell rang, the kid told me about feeling the pressure of schoolwork… and high expectations of parents… saying ‘I’m so exhausted, I can’t even breathe because of the pressure.’”*

(SN7)

School nurses pointed out that the background of this academically demanding environment is a structure where a school’s standing within the community depends on how many students it sends to good universities. Consequently, they have asserted that cases pertaining to at-risk students are often regarded as matters that should be concealed rather than disclosed within the academic environment. They further contend that the disclosure of such cases is frequently perceived as a problematic element that results in an increase in workload.

*“The school’s reputation is most strongly determined by how many students have gone on to top universities, not by how well it takes care of its students… From the perspective of school administrators, getting students into good universities is the primary goal! The ultimate goal! Caring for the kids inevitably has to take a backseat. As far as the school is concerned, just making sure no problems arise… Crisis management isn’t really the school’s main goal, is it?”*

(SN5)

Theme 11. School’s Dichotomous Approach to Students’ Physical and Mental Health

School nurses pointed out the shortcomings of the current school administrative system in supporting at-risk students. Within the context of school administration, the management of physical health concerns is typically the purview of the school nurse, while the handling of emotional well-being issues is often delegated to the school counselor. The system was structured so that the counselor would meet with the parents of students in crisis.

*“Currently, counselors handle cases where students are at risk of suicide, school nurses manage sexual matters, and the student affairs office takes charge when school violence incidents occur.”*

(SN1)

*“The school nurse treats physical issues or helps students with self-harm marks… Physical issues are handled by me (the school nurse), while emotional issues are handled by the counselor… There is only one child… One person. The child’s physical and mental issues are handled separately.”*

(SN6)

In this reality, school nurses stated that the school nurse’s office is often the first place students visit when facing difficulties. According to school nurses’ accounts, students were perceived to encounter challenges in accessing the school nurse for discussion regarding their difficulties. The perception of a distinction in which counseling falls under the purview of the counselor was described as creating barriers. Additionally, while school nurses acknowledged their capacity to provide a listening ear to students, they expressed uncertainty regarding the extent to which this role genuinely offers substantial support.

*“School nurses only make referrals since there’s a counselor available; they don’t actually conduct counseling themselves… Now we only handle basic wound care… Since the child usually comes to the school nurse’s office first when they’re struggling, I usually get a sense of the situation… After that, the counselor will take over.”*

(SN8)

*“Some kids just sit in the school nurse’s office and cry. Some kids go to counseling when I suggest it, while others refuse. If they say no, I ask, ‘Would it help if I just listened?’ and then I listen to their concerns. But since I can’t really help much, I find it quite difficult.”*

(SN2)

Theme 12. Lack of Communication Among Teachers in Crisis Response

Within the school’s crisis student management framework, collaboration among homeroom teachers, school nurses, and counselors was considered important. However, in practice, the implementation of cooperation is often perceived to be hindered by factors such as overwhelming workloads and divergent perspectives. Some school nurses and counselors expressed the perception that certain duties were not their responsibility, and in some cases, guidance was shared during teacher training sessions advising against taking on specific tasks.

*“Before the counselor system was introduced, the school nurse administered the Emotional and Behavioral Assessment. When the counselor was introduced, counseling for at-risk students became part of the counselor’s duties… Since the counselor system didn’t exist from the beginning, conflicts often arose due to improper division of responsibilities.”*

(SN5)

*“Some homeroom teachers think of at-risk students as ‘my students, so why are you getting involved?’ while others say, ‘It would be good if we all showed interest and cared for them together,’ and discuss such students whenever the need arises… However, we absolutely must not take a strict, dichotomous approach that compartmentalizes this strictly as work!”*

(SN4)

Theme 13. Excessive workload of School Nurses Limiting Student Support

School nurses, who are assigned to supervise the health of the entire student body, have reported experiencing a considerable workload burden as they are responsible for overseeing health classes and a variety of administrative tasks.

*“These days, the education office keeps piling on demands, so I really wonder if I should keep doing this. The workload itself is really heavy. The workload is insane. Even though it’s extracurricular, I work nonstop unlike the popular belief. I literally have no time to breathe, barely making it to the bathroom once in the morning and once in the afternoon…”*

(SN7)

In this environment of excessive workload, school nurses have stated that they find it difficult to allocate sufficient time to directly support and care for at-risk students.

*“If there’s just one troubled youth, it might be okay. There are many students with various problems, right? If student visits the school nurse’s office during my class hours, they must return to their classroom. Due to the risk of self-harm, I cannot leave them alone in the school nurse’s office without my supervision.”*

(SN2)

*“Sometimes when too many students come to the school nurse’s office, I do wonder, “Where exactly is the limit of our responsibilities?” There are times when I think, ‘Do we really have to manage that too?’ My responsibilities keep growing, and it seems like there’s more and more to do.”*

(SN8)

### 3.3. Triangulation of Quantitative and Qualitative Findings

**Figure 1:**
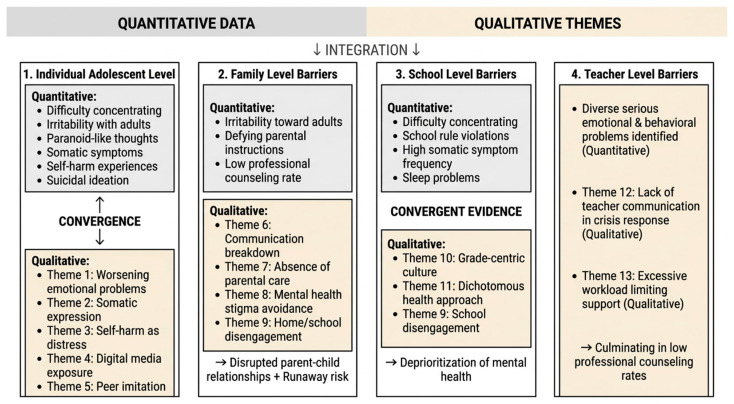
Triangulation of Quantitative and Qualitative Findings on Mental Health Barriers Among at-risk Adolescents.

The results of the triangulation of the quantitative and qualitative findings are presented in Figure 1.

First, At the Individual Adolescent Level: Worsening Emotional and Behavioral Problems and Inappropriate Modes of Expression

The difficulties in school life and interpersonal relationships identified in the quantitative data—difficulty concentrating (68.1%), irritability when given directions by adults (57.4%), the feeling that others know one’s thoughts (38.3%), and the feeling that others are gossiping about oneself (34.0%)—directly converged with the qualitative theme of Theme 1 (Worsening adolescent emotional and behavioral problems). In addition, the quantitative findings of somatic symptoms (46.8%), sleep difficulties (40.4%), and auditory hallucination-like experiences (23.4%) were associated with Theme 2 (Somatic symptoms as a means of expressing emotional distress), suggesting that emotional stress not articulated through language may be expressed indirectly through somatic and perceptual channels. A similar pattern was observed in self-harm and suicide-related responses: self-harm experience (12.8%), suicidal ideation (19.1%), concrete suicide planning (10.6%), and the belief that “things would be better if I were dead” (17.0%) were found to share a common context with Theme 3 (Self-harming behaviors as a means of expressing distress). Meanwhile, the quantitative findings of excessive use of the internet, gaming, and smartphones (44.7%) and alcohol consumption (21.3%) were conceptually linked to Theme 4 (Digital media as a source of harmful exposure) and Theme 5 (Peer imitation and reinforcement of crisis behaviors). That is, excessive use of digital media appears to function both as a means by which at-risk adolescents avoid emotional distress and as a medium through which crisis behaviors are learned, while substance use such as alcohol consumption may be sustained through peer-group imitation mechanisms.

Second, Barriers at the Family Level: Disrupted Parent–Child Relationships and the Risk of Running Away

The quantitative responses of irritability when given directions by adults (57.4%) and defying parental instructions (21.3%) could be related to the qualitative themes of Theme 6 (Communication breakdown between at-risk adolescents and their parents), Theme 7 (Absence of parental care in at-risk adolescents), and Theme 8 (Parents and adolescents reluctant to face mental health stigma). Furthermore, the low rate of professional counseling experience (25.5%) identified in the quantitative data could be linked to Theme 9 (Disengagement from home and school in at-risk adolescents).

Third, Barriers at the School Level: Deprioritization of Mental Health

The quantitative findings of difficulty concentrating (68.1%), serious violations of school rules such as truancy and running away (12.8%), the high frequency of somatic symptoms (46.8%), and sleep problems (40.4%) were connected with Theme 9 (Disengagement from home and school in at-risk adolescents), Theme 10 (Grade-centric school culture), and Theme 11 (School’s dichotomous approach to students’ physical and mental health). In other words, adolescents’ academic maladjustment and school disengagement appear to be pushed down the order of priority without receiving sufficient attention, within a grade-centric school culture and an administrative structure that addresses physical and mental health in a fragmented manner.

Fourth, Barriers at the Teacher Level: Low Rates of Professional Counseling Among At-Risk Adolescents

The diverse and serious emotional and behavioral problems of at-risk adolescents identified in the quantitative data of this study, when considered together with Theme 12 (Lack of communication among teachers in crisis response) and Theme 13 (Excessive workload of school nurses limiting student support) derived from school nurses, were ultimately found to culminate in the low rate of professional counseling experience (25.5%).

## 4. Discussion

Drawing on self-reported data from runaway adolescents and proxy reports from school nurses concerning at-risk adolescents in school settings, this study revealed that runaway adolescents represent one of the most acute manifestations of the broader crisis observed across the population of at-risk adolescents. The triangulation of these two data sources, therefore, was not intended to establish identity between the two populations, but rather to illuminate how the lived experiences of runaway adolescents as a subgroup intersect with the contextual realities of at-risk adolescents as a whole. The crisis behaviors of the participants in this study were found to be triggered and sustained by various barriers operating across the ecological systems of family, school, and community, and existing response systems were insufficient to address these issues.

Adolescents are predisposed to encountering mental health challenges, ranging from minor to major, due to their proclivity for risk-taking, egocentric reasoning, and heightened sensitivity to social pressures (Pfeifer & Berkman, 2018; Steinberg, 2010). From adolescence through early adulthood, behavior gradually changes and develops into more fully formed patterns (Roberts & DelVecchio, 2000), making this period a critical window during which mental health recovery and timely intervention are possible (Povey et al., 2025). The findings of this study indicated that runaway adolescents demonstrated relatively elevated rates of negative emotional and behavioral characteristics, including difficulty concentrating, irritability toward adults, paranoid-like ideation, somatization, sleep disturbances, self-harm, and suicidal ideation. These results align with prior research (Gross, 2015) indicating that individuals experiencing emotional stress are prone to difficulties in self-regulation and impulse control.

Notably, although adolescents exhibit a high prevalence of mental health conditions, they tend to show a comparatively low willingness to seek help or engage in treatment (Radez et al., 2021). Rather than directly articulating their depression or anxiety to others, they often express their distress through somatic symptoms such as recurrent stomachaches or headaches (Lu et al., 2025; Mahirah et al., 2025), and some respond by self-regulating their emotions or punishing themselves through self-harm—the deliberate damaging of one’s own body (Favazza, 1998; Klonsky, 2009). The triangulation of quantitative and qualitative data in this study revealed that somatic symptoms, sleep difficulties, and self-harming behaviors among runaway adolescents converged with the qualitative themes of “somatic symptoms as a means of expressing emotional distress” and “self-harming behaviors as a means of expressing distress.”

Adolescents also tend to seek similarity in peer relationships and to fortify social conformity (Laursen & Veenstra, 2021; Sherman et al., 2016). Martínez et al. (2023) explained that behavior and emotion spread within peer groups through social contagion (Martínez et al., 2023), and the imitation of adult risk behaviors can be interpreted as a manifestation of peer influence whereby adolescents conform to the behaviors of their peers (Laursen & Veenstra, 2021). In this study, school nurses identified somatization and self-harm as characteristic behaviors among at-risk adolescents and reported that the imitation and justification of crisis behaviors—such as peer smoking, drinking, and aggressive actions—are frequently observed in school settings. The high rates of excessive digital media use and alcohol consumption identified in the quantitative data are consistent with these observations, suggesting that digital media functions both as a means of avoiding emotional distress and as a medium through which crisis behaviors are learned and reinforced (Khalaf et al., 2023; Kysnes et al., 2022).

Steinberg (2007) posited that adolescent risky behaviors do not stem from simple irrationality but rather from a developmental imbalance between increased sensation-seeking urges and immature cognitive control abilities, and emphasized that modifying the environmental and contextual factors giving rise to risky behaviors yields more efficacious outcomes than focusing solely on individuals’ cognitive patterns (Steinberg, 2007). From this perspective, crisis behaviors such as self-harm and somatization should be understood not only as indicators of adolescents’ unstable emotional regulation, but also as early warning signs of underlying mental health concerns (Bohman et al., 2010; Klonsky, 2009; Lu et al., 2025).

Meanwhile, the significance of parental support for emotional stability and social adaptation during adolescence is well established. Parental emotional support and behavioral guidance have been shown to positively influence the development of emotional stability and self-esteem in adolescents, and these factors have been identified as crucial in preserving their mental health (Mackova et al., 2022; Xu & Ren, 2025). Ding et al. (2022) reported that the parent–child relationship is closely associated with adolescents’ mental health and that parental support can play a pivotal role in preventing depression, anxiety, and stress (Ding et al., 2022). Conversely, adolescents who spend considerable time in isolation without parental supervision become more susceptible to peer influence, thereby increasing their risk of engaging in antisocial behaviors. This risk is particularly exacerbated when insufficient parental supervision is combined with antisocial peer relationships (Nanninga et al., 2015; Sentse et al., 2010). The triangulation findings of this study showed that runaway adolescents’ irritability toward adults and defiance of parental instructions converged with the qualitative themes of “communication breakdown between at-risk adolescents and their parents,” “absence of parental care in at-risk adolescents,” and “parents and adolescents reluctant to face mental health stigma.” According to the Fragile Families and Child Wellbeing Study, unemployment and workplace inflexibility have been identified as factors that generate parental caregiving stress (Nomaguchi & Johnson, 2016). The present study suggested that when caregiving gaps emerge in households due to parents’ preoccupation with their livelihoods, adolescents’ crisis behaviors may be further exacerbated through peer interactions. This phenomenon aligns with prior research (Benatov, 2019; M.-H. Kim & Seo, 2017; Shalev et al., 2023) indicating that elevated parental stress levels are associated with a stronger tendency to attribute adolescents’ problems to external factors.

In environments where adequate parental care is not provided, adolescents are more likely to rely on self-harming behaviors as a means of coping with their negative emotions (Janssens et al., 2023). Conversely, studies have shown that when parents are able to manage their own stress and develop effective caregiving skills, significant reductions are observed in their children’s anxiety, depression, and behavioral problems (Adabla et al., 2024; Bakoula et al., 2009). Therefore, supporting at-risk adolescents requires a family ecosystem that strengthens parents’ emotional recovery and caregiving competence (Mackova et al., 2022).

Schools, where adolescents spend the greatest portion of their day, should regard mental health as a task no less important than academic achievement (Aspeqvist et al., 2024). However, the Korean school environment, which places excessive emphasis on college entrance examinations, continues to impose substantial academic pressure on adolescents, thereby fostering an environment in which serious mental health concerns—such as depression, anxiety, self-harm, and suicide—readily emerge (S. J. Kim et al., 2009; Suoniemi et al., 2021). In the present study, school nurses likewise reported that students were experiencing various forms of psychological crisis due to excessive academic stress, and stated that the priorities of school administration are concentrated on managing the institution’s external image, while support for students in crisis remains comparatively limited. The findings of this study showed that runaway adolescents’ difficulty concentrating, school rule violations, somatic symptoms, and sleep problems converged with the qualitative themes of “disengagement from home and school in at-risk adolescents,” “grade-centric school culture,” and “school’s dichotomous approach to students’ physical and mental health.” To address adolescent mental health concerns, the Korean Ministry of Education launched the Wee Project, establishing counseling rooms and assigning counselors in each school (Seo et al., 2014). However, students who experienced Wee Class counseling often feared social stigma and frequently held negative perceptions regarding the usefulness of counseling (Jeun & An, 2024). Prior studies have similarly reported that adolescents’ and parents’ concerns about stigma and their distrust of mental health services act as barriers to professional psychiatric counseling and treatment for at-risk adolescents (Lee et al., 2005; Owens et al., 2002). These findings indicate that for school-based mental health services to function effectively, the structural barriers within the ecosystem that hinder service utilization must be considered together. For example, this study revealed that the dichotomous administrative structure—in which “physical problems are handled by the school nurse” and “psychological problems are handled by the counselor”—functions as a barrier that deters stigma-fearing adolescents from using the counseling room, with a pattern emerging in which students used the school nurse’s office as a refuge under the pretext of physical symptoms. These findings suggest that moving beyond the dichotomous administrative separation within schools and introducing an integrated school health policy for promoting students’ mental health is critically important.

Furthermore, the findings of this study showed that the diverse and serious emotional and behavioral problems of runaway adolescents identified in the quantitative data, when considered in conjunction with the qualitative themes of “lack of communication among teachers in crisis response” and “excessive workload of school nurses limiting student support” derived from school nurses, were ultimately connected to the strikingly low rate of professional counseling experience (25.5%) reported by runaway adolescents. School nurses are positioned to be the first to detect early signs of changes in students’ daily behavior or physical and emotional distress (McDermott et al., 2018; Moen & Jacobsen, 2022; Thomas et al., 2025). In this study, school nurses recognized that self-harm and recurrent complaints of pain represent expressions of emotional distress, and were aware of the importance of appropriately identifying such signals. Therefore, supporting adolescents who are reluctant to directly express their emotional and behavioral issues requires teachers to exercise a high level of sensitivity, to collaborate closely with one another, and to maintain ongoing communication with parents (Hilli & Pedersen, 2021; Warren et al., 2024).

### Limitations and Strengths

The significance of this study lies in that, first, it directly engaged runaway adolescents, a population that has been difficult to recruit in academic research, as primary respondents, capturing their emotional and behavioral characteristics at the item level. Second, drawing on the field reality that at-risk adolescents often use the school nurses’ office as a refuge, this study employed school nurses as proxy informants who can illuminate the contextual realities of at-risk adolescents. Third, this study identified how various barriers to mental health support across the individual, family, school, and staff levels, including disrupted parent–child relationships, mental health stigma, and academically focused school cultures, contribute to the crises faced by high-risk adolescents.

A methodological consideration of this study lies in the asymmetry between the two data sources. The quantitative data were collected directly from runaway adolescents, whereas the qualitative data reflected school nurses’ observations of at-risk adolescents in general, including runaway adolescents. Although runaway adolescents are conceptually positioned in this study as a subgroup of at-risk adolescents who exhibit the most acute manifestations of runaway-related crises, the two populations are not strictly identical. Therefore, the convergences identified through triangulation should be understood as meaningful intersections between the two perspectives rather than as causal explanations applicable uniformly to both populations. Because direct in-depth interviews with adolescents or their parents were difficult to conduct due to participants’ psychological vulnerability and various constraints, this study was unable to directly capture the experiences and perceptions of at-risk adolescents and their guardians. In addition, the analysis of this study was confined to the individual, family, school, and staff levels and did not directly address community-level barriers, which are a constituent element of ecological models. Future research should employ multifaceted data collection methods that incorporate larger and more diverse samples, longitudinal designs, and the perspectives of community-based informants, including at-risk adolescents, their guardians, and community mental health practitioners.

## 5. Conclusions

This mixed-methods study, drawing on self-reported data from runaway adolescents and qualitative interviews with school nurses regarding at-risk adolescents in school settings, examined barriers to mental health care at the individual, family, school, and teacher levels. The results indicated that adolescents’ emotional distress tends to be expressed indirectly through somatic and behavioral channels, and may remain underaddressed within fragmented support systems.

These findings suggest the need for efforts to raise awareness of mental health and reduce stigma, as well as family-oriented approaches that strengthen parent–child communication and caregiving capacity. In addition, it is necessary to improve the academically focused school culture and to establish a school-based foundation that enables the early recognition of warning signs of adolescents’ mental health crises and facilitates efficient referral to professional counseling when needed. Furthermore, given that school nurses are positioned to be the first to observe changes in students’ daily behavior or early signs of physical and emotional distress, strengthening their professional competencies, fostering communication among teachers, and introducing measures to alleviate their workload should also be considered.

## Figures and Tables

**Table 1:** Emotional and Behavioral Characteristics of Runaway Adolescents (*n* = 47).

No. *	Question	Response	
		Yes, *n* (%)	No, *n* (%)
26	Often been sick here and there for no apparent reason. (e.g., headache, stomachache, vomit, nausea, dizziness, etc.)	22 (46.8)	25 (53.2)
27	Suffered ostracism and ignorance by others and found it hard to bear.	12 (25.5)	35 (74.5)
28	Experienced difficulty handling daily tasks due to excessive use of the Internet, games, and smartphone (e.g., conflicts with parents, difficulty at school, etc.)	21 (44.7)	26 (55.3)
29	Had serious mood swings for no apparent reason.	22 (46.8)	25 (53.2)
30	Been restless or continued to fidget with hands and feet.	30 (63.8)	17 (36.2)
31	Experienced binge eating and vomited sporadically.	7 (14.9)	40 (85.1)
32	Felt everything was troublesome and boring.	20 (42.6)	27 (57.4)
33	Found it hard to concentrate during lessons, study, or reading books for a long time.	32 (68.1)	15 (31.9)
34	Committed a serious violation of rules. (e.g., skipping school, running away from home, going to adult entertainment establishments, etc.)	6 (12.8)	41 (87.2)
35	Worried before things actually happened.	33 (70.2)	14 (29.8)
36	Become excessively nervous and messed up tasks.	27 (57.4)	20 (42.6)
37	Experienced difficulty falling asleep, or woken up often during night which gave me a hard time.	19 (40.4)	28 (59.6)
38	Suffered unwanted and repetitive thoughts or images.	17 (36.2)	30 (63.8)
39	Experienced hearing something that others could not hear.	11 (23.4)	36 (76.6)
40	Had trouble understanding the lessons.	16 (34.0)	31 (66.0)
41	Found it hard to forget and get over hardships that I experienced in the past (e.g., incidents, accidents, separation from or death of a close acquaintance/family member).	13 (27.7)	34 (72.3)
43	Felt very tense when with others.	25 (53.2)	22 (46.8)
44	Failed to wait his/her turn and acted before thinking.	21 (44.7)	26 (55.3)
45	Felt as if others knew what I was thinking.	18 (38.3)	29 (61.7)
46	Engaged in a certain behavior compulsively and felt undue stress. (e.g., washing hands, checking something, counting numbers, etc.)	8 (17.0)	39 (83.0)
48	Become irritated when adults instruct me to do this and that.	27 (57.4)	20 (42.6)
50	Experienced losing my temper and causing trouble.	19 (40.4)	28 (59.6)
51	Suffered teasing or bullying (verbal abuse, online bullying, physical abuse) and found it hard to bear.	5 (10.6)	42 (89.4)
52	Thought that others were talking behind my back.	16 (34.0)	31 (66.0)
53	Felt like dying.	9 (19.1)	38 (80.9)
54	Felt depressed or irritated for no apparent reason.	20 (42.6)	27 (57.4)
55	Defied or opposed to my parents or teachers.	10 (21.3)	37 (78.7)
56	Thought that others may be watching me or even preparing to harm me.	4 (8.5)	43 (91.5)
57	Devised a detailed plan to kill myself at least once.	5 (10.6)	42 (89.4)
58	Felt like my death would be an improvement to my situation.	8 (17.0)	39 (83.0)
59	Have had thoughts of wanting to commit suicide.	9 (19.1)	38 (80.9)
60	Have smoked cigarettes.	7 (14.9)	40 (85.1)
61	Have consumed alcoholic drinks.	10 (21.3)	37 (78.7)
62	Have used drug substances prohibited by law.	1 (2.1)	46 (97.9)
63	Seriously attempted suicide, thought it might be just once.	0 (0.0)	47 (100.0)
64	Received professional counselling.	12 (25.5)	35 (74.5)

* Includes only the 36 items pertaining to emotional and behavioral characteristics, including suicide risk, drawn from the original 65-item version of the AMPQ-III-I.

**Table 2:** General Characteristics of School Nurses as Proxy Informants (*n* = 8).

ID	Gender	Age	Education Level	Years as a School Nurse	School Type †	School Size (No. of Students)
SN 1	Female	51	Graduate degree	22.6	General academic high school	587
SN 2	Female	46	Associate degree	13.0	Specialized vocational high school	169
SN 3	Female	42	Bachelor’s degree	2.5	Specialized vocational high school	509
SN 4	Female	58	Bachelor’s degree	36.3	General middle school	386
SN 5	Female	55	Graduate degree	20.5	General academic high school	538
SN 6	Female	54	Bachelor’s degree	15.5	General academic high school	590
SN 7	Female	47	Associate degree	4.6	General middle school	400
SN 8	Female	43	Graduate degree	8.0	General middle school	273

† High schools were categorized into general academic high schools, which primarily follow a university-preparatory curriculum, and specialized vocational high schools, which offer career-oriented programs. All middle schools were classified uniformly as general middle schools.

**Figure A1:**
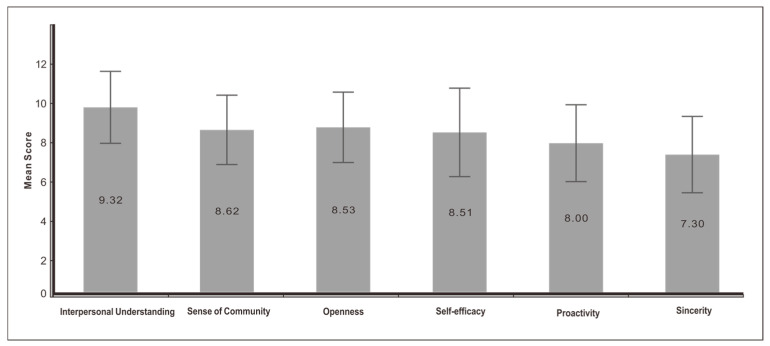
Personality Traits across Six Subscales Among Runaway Adolescents (*n* = 47). Error bars indicate standard deviation.

## Data Availability

The raw data supporting the conclusions of this article are not publicly available due to ethical and privacy restrictions but may be made available by the corresponding author upon reasonable request.

## Appendix A

The results of the Adolescent Mental Problem Questionnaire (AMPQ-III-I) administered to 47 runaway adolescents were as follows.

Among the subdomains of personality traits in runaway adolescents, the average score for interpersonal understanding (M = 9.32, SD = 2.79) was the highest, followed by sense of community (M = 8.62, SD = 2.69), openness (M = 8.53, SD = 2.73), self-efficacy (M = 8.51, SD = 3.43), and proactivity (M = 8.00, SD = 2.98). Sincerity (M = 7.30, SD = 2.96) showed the lowest average compared to other subdomains (Figure A1).

## Appendix B

1. How serious do you think the issue of at-risk adolescents is in our society today?
2. What do you think are the family factors that influence adolescent crisis behavior?
3. What do you think are the school factors that influence adolescent crisis behavior?
4. What do you think are the community factors that influence adolescent at-risk behavior?
5. What are the early warning signs or clues indicating an adolescent crisis?
6. What emotional or behavioral problems might at-risk adolescents exhibit?
7. How does the school intervene or provide support for emotional and behavioral issues among adolescents?
8. What were the obstacles (family, school, or community) you encountered in the process of supporting at-risk adolescents?
9. What are the outcomes of school-based interventions for at-risk adolescents?
10. If there are cases where, despite school-level interventions, at-risk adolescents ultimately run away from home or become out-of-school youth, please describe the process.

## References

[B1-behavsci-16-00833] Adabla S., Nabors L. A., Olaniyan A., Merianos A. (2024). Correlates of behavioral problems among youth with anxiety. Journal of Child and Family Studies.

[B2-behavsci-16-00833] Ahmed S. K. (2024). The pillars of trustworthiness in qualitative research. Journal of Medicine, Surgery, and Public Health.

[B3-behavsci-16-00833] Askari G., Vajdi M., Jafari-Nasab S., Golpour-Hamedani S. (2024). Ethical guidelines for human research on children and adolescents: A narrative review study. Journal of Research in Medical Sciences.

[B4-behavsci-16-00833] Aspeqvist E., Munger A. C., Andersson H., Korhonen L., Baetens I., Dahlstrom O., Zetterqvist M. (2024). Adolescents’ experiences of a whole-school preventive intervention addressing mental health and nonsuicidal self-injury: A qualitative study. BMC Public Health.

[B5-behavsci-16-00833] Bakoula C., Kolaitis G., Veltsista A., Gika A., Chrousos G. P. (2009). Parental stress affects the emotions and behaviour of children up to adolescence: A Greek prospective, longitudinal study. Stress.

[B6-behavsci-16-00833] Benatov J. (2019). Parents’ feelings, coping strategies and sense of parental self-efficacy when dealing with children’s victimization experiences. Frontiesr in Psychiatry.

[B7-behavsci-16-00833] Bohman H., Jonsson U., Von Knorring A. L., Von Knorring L., Paaren A., Olsson G. (2010). Somatic symptoms as a marker for severity in adolescent depression. Acta Paediatrica.

[B8-behavsci-16-00833] Braun V., Clarke V. (2006). Using thematic analysis in psychology. Qualitative Research in Psychology.

[B9-behavsci-16-00833] Castillo B., Schulenberg J., Grogan-Kaylor A., Toro P. A. (2023). The prevalence and correlates of running away among adolescents in the United States. Journal of Community Psychology.

[B10-behavsci-16-00833] Ding F., Jia Y., Xiong X., Chen P., Xiong S., Cheng G. (2022). The protective role of parental involvement at home in negative psychological outcomes among Chinese adolescents during the COVID-19 epidemic. Journal of Affective Disorders.

[B11-behavsci-16-00833] Favazza A. R. (1998). The coming of age of self-mutilation. The Journal of Nervous & Mental Disease.

[B12-behavsci-16-00833] Gross J. J. (2015). Emotion regulation: Current status and future prospects. Psychological Inquiry.

[B13-behavsci-16-00833] Hilli Y., Pedersen G. (2021). School nurses’ engagement and care ethics in promoting adolescent health. Nursing Ethics.

[B14-behavsci-16-00833] Janssens J. J., Myin-Germeys I., Lafit G., Achterhof R., Hagemann N., Hermans K., Hiekkaranta A. P., Lecei A., Kirtley O. J. (2023). Lifetime and current self-harm thoughts and behaviors and their relationship to parent and peer attachment. Crisis.

[B15-behavsci-16-00833] Jeun J.-H., An H.-Y. (2024). High school students’ perceptions of the intention to seek help from wee class school counselors: Effects of individual and environmental factors. The Korean Journal of School Psychology.

[B16-behavsci-16-00833] Khalaf A. M., Alubied A. A., Khalaf A. M., Rifaey A. A. (2023). The impact of social media on the mental health of adolescents and young adults: A systematic review. Cureus.

[B17-behavsci-16-00833] Kim E. H., Kim K. H. (2016). School counselors’ experiences of students with emotional and behavioral disabilities. Journal of Emotional & Behavioral Disorders.

[B18-behavsci-16-00833] Kim H. R., Moon S. H. (2023). Predictors for runaway behavior in adolescents in South Korea: National data from a comprehensive survey of adolescents. Frontiers in Psychiatry.

[B19-behavsci-16-00833] Kim J., Cho M. (2024). Path to suicidality in Korean adolescents: Mediating role of self-esteem, somatic symptoms, and self-harm amid depressive symptoms. Healthcare.

[B20-behavsci-16-00833] Kim M.-H., Seo J.-M. (2017). Parents’ knowledge and attitudes regarding a screening test for and subsequent management of students’ emotional and behavioral problems. Child Health Nursing Research.

[B21-behavsci-16-00833] Kim S. J., Lee C. S., Kweon Y. R., Oh M. R., Kim B. Y. (2009). Test of validity and reliability of the adolescent mental problem questionnaire for Korean high school students. Journal of Korean Academy of Nursing.

[B22-behavsci-16-00833] Kisiel C., Conradi L., Fehrenbach T., Torgersen E., Briggs E. C. (2014). Assessing the effects of trauma in children and adolescents in practice settings. Child and Adolescent Psychiatric Clinics of North America.

[B23-behavsci-16-00833] Klonsky E. D. (2009). The functions of self-injury in young adults who cut themselves: Clarifying the evidence for affect-regulation. Psychiatry Research.

[B24-behavsci-16-00833] Korea Disease Control and Prevention Agency (2025). The 2024 Korea youth risk behavior survey: Results report.

[B25-behavsci-16-00833] Korea Educational Environment Protection Agency (2025). 2025 Student emotional and behavioral characteristics screening test and management manual.

[B26-behavsci-16-00833] Kysnes B., Hjetland G. J., Haug E., Holsen I., Skogen J. C. (2022). The association between sharing something difficult on social media and mental well-being among adolescents. Results from the “LifeOnSoMe”-study. Frontiers in Psychology.

[B27-behavsci-16-00833] Laursen B., Veenstra R. (2021). Toward understanding the functions of peer influence: A summary and synthesis of recent empirical research. Journal of Research on Adolescence.

[B28-behavsci-16-00833] Lee Y.-S., Suh D.-S., Hong S.-D., An D.-H., Song D.-H., Kim B.-N. (2005). Help seeking behavior about children’s problem and satisfaction with psychiatric service: Multicenter point epidemiologic study. Journal of Korean Neuropsychiatric Association.

[B29-behavsci-16-00833] Lu J., Han Y., Liu X., Li W., Zhou X. (2025). Association between somatic symptoms and depression and anxiety in adolescents: A cross-sectional school-based study. BMJ Open.

[B30-behavsci-16-00833] Mackova J., Veselska Z. D., Geckova A. M., Jansen D., van Dijk J. P., Reijneveld S. A. (2022). The role of parents in the care for adolescents suffering from emotional and behavioral problems. Frontiers in Psychology.

[B31-behavsci-16-00833] Mahirah D., Lim J. M., Chew M. S., Peddapalli N., Ho C. Z., Marimuttu V. J., Chen H. Y., Sung S. C., Ho Y. L., Loh C. B. (2025). Prevalence and associated factors of somatic symptoms among adolescents in Singapore: A cross-sectional study. Annals of General Psychiatry.

[B33-behavsci-16-00833] Martinez-Casanova E., Molero-Jurado M. D. M., Perez-Fuentes M. D. C. (2024). Self-esteem and risk behaviours in adolescents: A systematic review. Behavioral Sciences.

[B32-behavsci-16-00833] Martínez V., Jimenez-Molina A., Gerber M. M. (2023). Social contagion, violence, and suicide among adolescents. Current Opinions on Psychiatry.

[B34-behavsci-16-00833] McDermott E., Bohnenkamp J. H., Freedland M., Baker D., Palmer K. (2018). The school nurse’s role in behavioral/mental health of students. Position statement. National Association of School Nurses.

[B35-behavsci-16-00833] Ministry of Gender Equality and Family (2025). 2025 Youth program guidelines.

[B36-behavsci-16-00833] Moen O. L., Jacobsen I. C. R. (2022). School nurses’ experiences in dealing with adolescents having mental health problems. SAGE Open Nursing.

[B37-behavsci-16-00833] Nanninga M., Jansen D. E., Knorth E. J., Reijneveld S. A. (2015). Enrolment of children and adolescents in psychosocial care: More likely with low family social support and poor parenting skills. European Child & Adolescent Psychiatry.

[B38-behavsci-16-00833] Nomaguchi K., Johnson W. (2016). Parenting stress among low-income and working-class fathers: The role of employment. Journal of Family Issues.

[B39-behavsci-16-00833] Owens P. L., Hoagwood K., Horwitz S. M., Leaf P. J., Poduska J. M., Kellam S. G., Ialongo N. S. (2002). Barriers to children’s mental health services. Journal of the American Academy of Child & Adolescent Psychiatry.

[B40-behavsci-16-00833] Palinkas L. A., Horwitz S. M., Green C. A., Wisdom J. P., Duan N., Hoagwood K. (2015). Purposeful sampling for qualitative data collection and analysis in mixed method implementation research. Administration and Policy in Mental Health and Mental Health Services Research.

[B41-behavsci-16-00833] Pfeifer J. H., Berkman E. T. (2018). The development of self and identity in adolescence: Neural evidence and implications for a value-based choice perspective on motivated behavior. Child Development Perspectives.

[B42-behavsci-16-00833] Povey J., Austerberry S., Plage S., Xiang N., Bellotti M., Baxter J. (2025). Adolescent mental health and parent engagement in secondary school: A systematic review of the literature. International Journal of Educational Research.

[B43-behavsci-16-00833] Prince D. M., Vidal S., Okpych N., Connell C. M. (2019). Effects of individual risk and state housing factors on adverse outcomes in a national sample of youth transitioning out of foster care. Journal of Adolescence.

[B44-behavsci-16-00833] Radez J., Reardon T., Creswell C., Lawrence P. J., Evdoka-Burton G., Waite P. (2021). Why do children and adolescents (not) seek and access professional help for their mental health problems? A systematic review of quantitative and qualitative studies. European Child & Adolescence Psychiatry.

[B45-behavsci-16-00833] Rickwood D., Deane F. P., Wilson C. J., Ciarrochi J. (2005). Young people’s help-seeking for mental health problems. Australian e-Journal for the Advancement of Mental Health.

[B46-behavsci-16-00833] Roberts B. W., DelVecchio W. F. (2000). The rank-order consistency of personality traits from childhood to old age: A quantitative review of longitudinal studies. Psychology Bulletin.

[B47-behavsci-16-00833] Santelli J. S., Ott M. A., English A., Sawyer S. M., Sieving R. E., Nagata J. M., Melamed I., Rosenfeld W. D., Rosen G., Ssewamala F. M. (2025). Guidelines on the inclusion and protection of adolescent minors and young adults in health research: A position statement of the society for adolescent health and medicine. Journal of Adolescence Health.

[B48-behavsci-16-00833] Sappenfield O., Alberto C., Minnaert J., Donney J., Lebrun-Harris L., Ghandour R. (2024). National survey of children’s health: Adolescent mental and behavioral health, 2023. National survey of children’s health data briefs.

[B49-behavsci-16-00833] Schnyder N., Panczak R., Groth N., Schultze-Lutter F. (2017). Association between mental health-related stigma and active help-seeking: Systematic review and meta-analysis. The British Journal of Psychiatry.

[B50-behavsci-16-00833] Sentse M., Dijkstra J. K., Lindenberg S., Ormel J., Veenstra R. (2010). The delicate balance between parental protection, unsupervised wandering, and adolescents’ autonomy and its relation with antisocial behavior: The TRAILS study. International Journal of Behavioral Development.

[B51-behavsci-16-00833] Seo G. S., Lee H. S., Goak Y. J., Kwon Y. H., Oh M. S. (2014). The perceptions of wee project for supporting students with emotional and behavioral disorders. Journal of Emotional & Behavioral Disorders.

[B52-behavsci-16-00833] Shalev I., Sharon N., Uzefovsky F., Atzaba-Poria N. (2023). Parental guilt and children’s internalizing and externalizing behavior: The moderating role of parental reflective functioning. Journal of Family Psychology.

[B53-behavsci-16-00833] Sherman L. E., Payton A. A., Hernandez L. M., Greenfield P. M., Dapretto M. (2016). The power of the like in adolescence: Effects of peer influence on neural and behavioral responses to social media. Psychological Science.

[B54-behavsci-16-00833] Sikand A., Schubiner H., Simpson P. M. (1997). Parent and adolescent perceived need for parental consent involving research with minors. Archives of Pediatrics & Adolescent Medicine.

[B55-behavsci-16-00833] Steinberg L. (2007). Risk taking in adolescence: New perspectives from brain and behavioral science. Current Directions in Psychological Science.

[B56-behavsci-16-00833] Steinberg L. (2010). A dual systems model of adolescent risk-taking. Developmental Psychobiology.

[B57-behavsci-16-00833] Suoniemi S., Rantanen A., Koivisto A. M., Joronen K. (2021). Self-reported school difficulties and the use of the school nurse services by adolescent students. Children.

[B58-behavsci-16-00833] Thomas C. S., Nielsen T. K., Best N. C. (2025). A rapid review of mental health training programs for school nurses. The Journal of School Nursing.

[B59-behavsci-16-00833] Wanniarachchi V. U., Greenhalgh C., Warren J. (2025). Adolescents’ and youths’ perceived barriers and facilitators to engaging with digital mental health interventions for depression and anxiety: A scoping review. Internet Interventions.

[B60-behavsci-16-00833] Warren J. M., Blount T. N., Belle G. (2024). Implementing effective school-based mental health services: A guide for school counselors. Professional School Counseling.

[B61-behavsci-16-00833] WHO (n.d.). Global strategy for women’s, children’s and adolescents’ health data portal.

[B62-behavsci-16-00833] World Medical Association (2025). World Medical Association Declaration of Helsinki: Ethical principles for medical research involving human participants. JAMA.

[B63-behavsci-16-00833] Xu T., Ren J. (2025). Parent-child relationships, parental control, and adolescent mental health: An empirical study based on ceps 2013–2014 survey data. Behavioral Sciences.

[B64-behavsci-16-00833] Zhang J.-Y., Zhang H., Chen Y., Zhang L.-H., Zhou Y., Li Y. (2025). Parental neglect and social media addiction of adolescents: The chain mediation effect of basic psychological need and personal growth initiative. Journal of Pediatric Nursing.

